# To Correspondents

**Published:** 1845-09

**Authors:** 


### TO CORRESPONDENTS.

We have received communications from Doctors Bailey and Turnbull, which shall appear in our next number.

